# Using AI Text-to-Image Generation to Create Novel Illustrations for Medical Education: Current Limitations as Illustrated by Hypothyroidism and Horner Syndrome

**DOI:** 10.2196/52155

**Published:** 2024-02-22

**Authors:** Ajay Kumar, Pierce Burr, Tim Michael Young

**Affiliations:** 1 Queen Square Institute of Neurology University College London London United Kingdom

**Keywords:** artificial intelligence, AI, medical illustration, medical images, medical education, image, images, illustration, illustrations, photo, photos, photographs, face, facial, paralysis, photograph, photography, Horner's syndrome, Horner syndrome, Bernard syndrome, Bernard's syndrome, miosis, oculosympathetic, ptosis, ophthalmoplegia, nervous system, autonomic, eye, eyes, pupil, pupils, neurologic, neurological

## Abstract

Our research letter investigates the potential, as well as the current limitations, of widely available text-to-image tools in generating images for medical education. We focused on illustrations of important physical signs in the face (for which confidentiality issues in conventional patient photograph use may be a particular concern) that medics should know about, and we used facial images of hypothyroidism and Horner syndrome as examples.

## Introduction

Artificial intelligence (AI) has become integral in medicine, outperforming skilled radiologists in certain domains [1]. However, there is limited exploration of AI's potential in producing illustrations for medical education [2,3]. Confidentiality concerns can limit traditional patient photo use, especially when facial features are essential [4]. Using widely available AI text-to-image tools, we aimed to create images portraying distinct facial signs important for medical trainees—hypothyroidism (myxedema) and Horner syndrome [5,6]. These tools generate unique, high-quality images based on text prompts, utilizing learned probability distributions rather than pre-existing images [7].

## Methods

ChatGPT was used to generate prompts for the two AI text-to-image tools used in this study—DALL·E 2 and Midjourney (Multimedia Appendix 1) [8-10], with which the prompts were used to generate images for hypothyroidism and Horner syndrome. The images were assessed and selected, using the following suitability criteria:

1. Images were excluded if any of the following features were present: insufficient coverage of the face, blurred images, a lack of realistic or humanoid features, a lack of continuity of edges, background noise, cloning errors, and geometrical and shadow inconsistencies.
2. Remaining images were accepted if they adequately represented the facial features of hypothyroidism or Horner syndrome, as judged by the coauthors (all were experienced physicians).

If adequate images could not be generated via the above methods, additional prompts, which were not generated with ChatGPT, were used. If adequate images were still not generated, then secondary editing via Microsoft Paint and GNU Image Manipulation Program (GIMP) was performed on the best image to try and meet the criteria listed above.

## Results

### Facial Features of Hypothyroidism

Using ChatGPT, the following text prompt was generated (restricted to the DALL·E 2 prompt word limit):

Generate an image depicting a middle-aged Caucasian woman with hypothyroidism presenting with facial myxedema. The woman should be shown in a frontal view, focusing on her face, scalp, and neck, without any makeup. The face must be very rounded and extreme scalp balding with coarse hair. Skin looks dry and pale. Outer eyebrows have a paucity of hairs, eyelids look very puffy. She looks tired.

**Figure 1:**
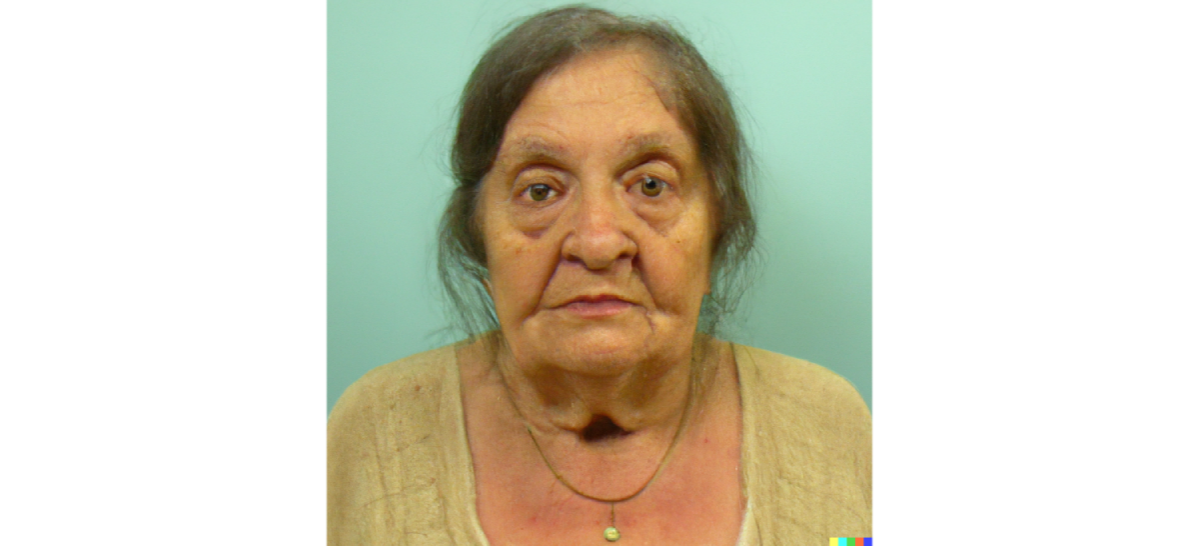
Artificial intelligence text-to-image production of facial features typical of hypothyroidism (myxedema) showing classical clinical features, including a rounded face with dry, pale skin; puffy eyelids; a general appearance of tiredness; and partial balding with coarse hair and loss of hair in the eyebrows (especially in the outer third). This image was produced by using DALL·E 2 [9] alone and without additional editing.

The prompt was used to generate 120 images. Of these, 53 were removed, using our preset exclusion criteria. Of the remaining 67, only 17 met some of the criteria for adequately representing facial features of hypothyroidism. The best image was selected as Figure 1 [9], with no additional editing needed.

### Horner Syndrome

The following prompt was obtained from ChatGPT:

Create an illustrative depiction of a patient displaying Horner's syndrome, emphasizing the key clinical features, such as ptosis (drooping of the upper eyelid), miosis (constricted pupil), and anhidrosis (lack of sweating) on one side of the face. Ensure the image is clear and medically accurate, aiding in the understanding of this neurological condition.

**Figure 2:**
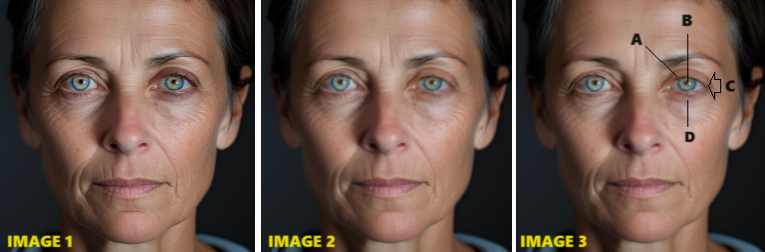
Generated illustration of Horner syndrome. Image 1 was produced by using Midjourney [10]. Image 2 shows the result after minor image editing (as described in our *Methods* section) to attenuate the key teaching features, which are labeled in image 3 (A: ptosis; B: miosis; C: apparent enophthalmos; D: upside-down ptosis).

Of the 120 images, 85 met our exclusion criteria, but none met our inclusion criteria, even after alternative prompts and DALL·E 2 were used. We therefore selected the best image (produced by Midjourney) and then performed secondary editing with Microsoft Paint and GIMP (Figure 2 [10]). This produced an image of Horner syndrome that was judged as adequate.

## Discussion

We aimed to explore the potential, as well as the current limits, of AI text-to-image generation in producing illustrations of medical conditions affecting the face. Without the use of high-quality medical images, it can be more challenging to teach others about these important conditions [11]. We showed that AI text-to-image generation is readily possible for hypothyroidism—a condition with symmetrical features. However, for Horner syndrome—a condition with asymmetrical features—adequate images could only be produced after some additional slight editing, reflecting a possible limiting factor of these tools. Ours are the first AI-generated images of classical facial features of hypothyroidism and Horner syndrome that we are aware of.

Confidentiality has become an increasing concern in the use of medical images over the last few decades. Text-to-image tools have ethical issues, including issues of consent for the original photos used to train these tools. Additionally, issues of accuracy are key. Nonmedics might be misled on medical signs by using such tools. Targets for future research are the potential for biases with these tools and the danger of stereotypes being perpetuated. Despite these limitations, AI-generated images may enhance case-based learning, allowing students to study and analyze a diverse range of medical cases. Text-to-image tools show exciting potential and may allow easier access to high-quality images in medical education [12,13].

## Appendix

Multimedia Appendix 1 Tools used in this article (all prompts entered in English).

## Abbreviations

AI: artificial intelligence
GIMP: GNU Image Manipulation Program
