# Supplementary material for: Using AI Text-to-Image Generation to Create Novel Illustrations for Medical Education: Current Limitations as Illustrated by Hypothyroidism and Horner Syndrome
Source: JMIR Med Educ. 2024 Feb 22;10:e52155. doi: 10.2196/52155 (PMC10921331; doi:10.2196/52155)
Supplement: Multimedia Appendix 1 [file mededu_v10i1e52155_app1.docx]

**Tools used in this article (all prompts entered in English):**

ChatGPT 3.5: Text generated by ChatGPT August version 2023, OpenAI, https://chat.openai.com. ChatGPT.

DALL·E 2: images generated by DALL·E 2 July-December 2023, OpenAI, <https://openai.com/dall-e-2>.

Midjourney alpha iteration of version 5 Publisher Midjourney via Discord URL of the AI system. <https://www.midjourney.com/home/>

Other tools described in the text:

Microsoft Paint version21H2 (OS Build 19044.3803)

Gnu Image Manipulation Program (GIMP) (version GIMP 2.10.34.) https://www.gimp.org/
